# Supplementary material for: Doubting what you already know: Uncertainty regarding state transitions is associated with obsessive compulsive symptoms
Source: PLoS Comput Biol. 2020 Feb 27;16(2):e1007634. doi: 10.1371/journal.pcbi.1007634 (PMC7046195; doi:10.1371/journal.pcbi.1007634)
Supplement: S1 Text — (DOCX) [file pcbi.1007634.s001.docx]

**S1 Text. Procedure and results of the Posner task**

The Posner task was similar to the alternative forced choice (AFC) task reported in the paper, with the exception that instead of predicting the location of the target by the arrow keys, participants were asked to respond as quickly as possible to the appearance of the target by using the space bar. Participants were given similar instructions, while being explicitly told that paying attention to the feedback to track the relevant cue can help them respond faster.

The analysis of response time (RT) is known to be sensitive to outliers, which may result from various causes, such as: lapses of attention leading to slow RTs, slips of action leading to fast RTs, and more [1,2]. To remove outliers, we first removed all RTs below 1ms (implying a response that preceded the target's appearance; resulting in a removal of 3.72% of the trials). Then, we followed the *modified recursive method* suggested by Van Selst and Jolicoeur [2]. This method has several potential advantages. First, it chooses the cutoffs (Z scores) for removal based on the number of trials per participant – which is important given the evidence for the sensitivity of outlier removal results to the number of trials [3]. Second, instead of choosing a single cutoff (RT derived from the chosen Z score) based on the entire RT distribution (per participant), it recalculates the mean and the standard deviation determining the RTs used as cutoffs after each removal of an extreme observation. This procedure is less sensitive to very extreme outliers which otherwise bias the mean and standard deviation used for converting the Z-score cutoff to raw RT.

After removing outliers using this method (resulting an overall removal of 6.70% of the trials), we first examined whether there is sufficient evidence for a validity effect. This effect can be tested at several levels. First, one can use all trials to examine whether participants respond faster on valid trials. However, even a participant who is not trying to find the relevant cue can exhibit an apparent validity effect driven solely by the number of cues pointing at a certain direction. Thus, for example, when all three cues point right, the expectation (and potential violation thereof) that the target will appear to the right will likely lead to a validity effect, regardless of whether the participant learns at all. Instead, we focused on a subset of trials in which the relevant cue pointed at one direction, and the other two cues pointed at the opposite direction. If found - a validity effect in these trials will provide clear evidence for learning.

The results suggested evidence for a validity effect only pre-shift (*t =* -2.18, *p* = .031), but not post-shift (*t =* -0.66, *p* = .512). However, these results should be taken with extreme caution, as running the analysis without the removal of outliers (other than RTs < 1ms) lead to non-significant results (pre-shift: *t =* -0.56, *p* = .573, post-shift: *t* = -0.73, *p* = .456).

Next, we examined whether the validity effect found in the pre-shift block (after outlier removal) can be explained by information-theoretic measures of prediction errors (namely the KL-divergence between the prior and the posterior at each trial, and the surprisal). To obtain these measures, we used the BCP model with parameter estimates derived from the AFC task. The use of estimates derived from a different task renders the present analysis an examination of both the validity of RTs in this block (as a measure of prediction errors), and the out-of-sample predictive validity of the AFC parameter estimates. Critically, because KL-divergence and surprisal are highly affected by whether the trial was valid or not (i.e. invalid trials result in a higher prediction error), we controlled for (actual) validity when examining the effect of the KL-divergence on RTs. This examines whether the participant-specific measures of prediction errors explain RTs over and above the actual unpredictability of the target (i.e. whether it is valid or not). Linear multi-level models (with a random intercept and slopes) were used for this analyses.

This analysis showed that the KL-divergence had a significant effect on RTs in the pre-shift block (β = 15.21, *t* = 2.975, *p* = .005), whereas the effect of surprisal was not significant (β = 22.66, *t* = 1.64, *p* = .104). In both cases, the effect of actual validity became non-significant (β's ≥ -6.25, *t*'s ≥ -1.433, *p*'s ≥ .155). These results suggest that the validity effect found in the pre-shift block is fully explained by the extent to which participants update their model of the environment (i.e. the distribution over cues) following feedback, but not by a simple measure of the feedback unpredictability.

Finally, to examine whether OCI-R scores modulate the validity effect, we entered OCI-R scores, validity and their interaction as predictors of RTs in the pre-shift block. Whereas a trend for a positive relationship between OCI-R scores and RTs in this block was found (β = 1.18, *t* = 1.86, *p* = .068), the interaction between OCI-R and validity was not significant (β = -0.08, *t* = -0.27, *p* = .786). These results partially support the suggestion (in the main text) that although transition uncertainty is elevated in high OC participants, this does not lead to increased model updating as measured by the KL divergence (in the paper) and the validity effect reported here. In contrast, surprisal is affected by OC symptoms, but does not seem to underly the validity effect. We discuss the possible meaning of this pattern of results in the main text.

**References**

1. Ratcliff R. Methods for dealing with reaction time outliers. Psychological bulletin. 1993;114: 510.

2. Van Selst M, Jolicoeur P. A solution to the effect of sample size on outlier elimination. The Quarterly Journal of Experimental Psychology Section A. 1994;47: 631–650.

3. Miller J. Reaction time analysis with outlier exclusion: Bias varies with sample size. The quarterly journal of experimental psychology. 1991;43: 907–912.
