# Supplementary material for: Doubting what you already know: Uncertainty regarding state transitions is associated with obsessive compulsive symptoms
Source: PLoS Comput Biol. 2020 Feb 27;16(2):e1007634. doi: 10.1371/journal.pcbi.1007634 (PMC7046195; doi:10.1371/journal.pcbi.1007634)
Supplement: S2 Text — (DOCX) [file pcbi.1007634.s002.docx]

**S2 Text – Discussion of the full model comparison results**

S2 Table presents the full model comparison results, including all examined models. Only a subset of the (best-fitting) models examined in the probabilistic condition (S2A Table) where examined in the deterministic condition (marked by equivalent indices in S2B Table). This allowed to reduce the number of models, which was important since each model in the deterministic condition was examined both when fixing γ (marked with the letter 'd' next to the model index), and when allowing it to be estimated as a free parameter.

The rightmost part of the tables presents the WAIC differences in standard error units. These can be used to estimate whether two models are statistically different from each other. For example, a difference that is 2SEs or larger indicates that a classical confidence interval (±1.96SE) will not include zero. However, since studies suggest that SE estimates as those used by the loo package [1] can underestimate the true variance (up to a factor of 2; [2]), we refrain from categorizing model differences as significant. However, a difference that is less than 2SEs can be used to infer that the corresponding models are not significantly different than each other (since overestimation is unlikely).

The best-fitting models in the probabilistic condition (but not the deterministic condition) were models that included an inverse temperature (β) parameter, that were used to examine whether participants use a maximizing response style – i.e., overweighting the most likely cues. However, we decided not to use these models to examine individual differences (i.e. correlation with OCI-R scores) for several reasons. First, the difference between these models and more simple BCP models was negligible (0.7SEs in the probabilistic condition). Second, whereas participants tended to overweight the most likely cue, the magnitude of overweighting was small (median β = 1.67, 95% HDI [1.09, 2.56], where 1 indicates no overweighting, and values > 5 are required for a full maximizing response style). Third, the β parameter had identifiability issues since there was a strong negative correlation (between MCMC samples) between the (group-level) β and the γ parameters (r = -0.87). This tradeoff corresponds with the idea that low γ values can balance maximizing by increasing the variance of $p\left( c | D_{1:t} \right)$. That is, for β of 1 (indicating no maximizing) group-level γ was distributed around 0.75 (similar to models not including the β parameter, and to the actual cue validity). Conversely, for posterior β values corresponding with maximizing (~5), group-level γ was distributed around 0.55, which is less appropriate given the actual cue validity in the task. Furthermore, an extremely high correlation was found between the two parameters between participants as well (r = 0.85). This precludes the interpretation of the correlations between each parameter and other variables (e.g., OCI-R scores).

References

1. Vehtari A, Gelman A, Gabry J. Practical Bayesian model evaluation using leave-one-out cross-validation and WAIC. Stat Comput. 2017;27: 1413–1432. doi:10.1007/s11222-016-9696-4

2. Bengio Y, Grandvalet Y. No Unbiased Estimator of the Variance of K-Fold Cross-Validation. Journal of Machine Learning Research. 2004;5: 1089–1105.
