# Supplementary material for: Doubting what you already know: Uncertainty regarding state transitions is associated with obsessive compulsive symptoms
Source: PLoS Comput Biol. 2020 Feb 27;16(2):e1007634. doi: 10.1371/journal.pcbi.1007634 (PMC7046195; doi:10.1371/journal.pcbi.1007634)
Supplement: S1 Table — (DOCX) [file pcbi.1007634.s003.docx]

**S1 Table – Effects of OCI-R subscales on BCP model parameters (comparing changing *h* to constant *h* models)**

| Questionnaire score | Model parameter | Pearson r (permutation test p-value) | | | |
| --- | --- | --- | --- | --- | --- |
|  |  | Constant *h* model | | Changing *h* model | |
|  |  | Probabilistic | Deterministic | Probabilistic | Deterministic |
| OCI-R  *Total* | *h* | .31 (.017) | .24 (.062) | .34 (.009) | .27 (.038) |
|  | *1-*$\gamma$ | .15 (.259) | -.06 (.704) | .25 (.064) | -.03 (.836) |
|  | $\epsilon$ | .05 (.732) | .06 (.639) | .05 (.715) | .10 (.496) |
| OCI-R  Checking | *h* | .25 (.053) | .28 (.035) | .29 (.027) | .29 (.028) |
|  | *1-*$\gamma$ | .07 (.597) | -.05 (.730) | .15 (.274) | -.03 (.823) |
|  | $\epsilon$ | .06 (.662) | .04 (.791) | .06 (.649) | .10 (.452) |
| OCI-R  Neutralizing | *h* | .43 (.001) | .36 (.011) | .41 (.004) | .43 (.006) |
|  | *1-*$\gamma$ | .32 (.015) | -.05 (.720) | .41 (.002) | -.03 (.835) |
|  | $\epsilon$ | .28 (.030) | .18 (.158) | .29 (.030) | .20 (.132) |
| OCI-R  Hoarding | *h* | .37 (.004) | .24 (.070) | .39 (.002) | .28 (.034) |
|  | *1-*$\gamma$ | .11 (.425) | -.07 (.659) | .21 (.117) | -.03 (.859) |
|  | $\epsilon$ | .13 (.343) | .06 (.636) | .13 (.325) | .10 (.477) |
| OCI-R  Washing | *h* | .26 (.054) | .17 (.019) | .27 (.035) | .14 (.308) |
|  | *1-*$\gamma$ | .03 (.824) | -03 (.843) | .17 (.208) | -.04 (.920) |
|  | $\epsilon$ | .06 (.664) | .17 (.222) | .06 (.674) | .17 (.195) |
| OCI-R  Obsessing | *h* | -.01 (.905) | -.12 (.376) | .05 (.731) | .14 (.304) |
|  | *1-*$\gamma$ | .11 (.418) | -.02 (.868) | .10 (.431) | .01 (.920) |
|  | $\epsilon$ | -.12 (.364) | -.05 (.694) | -.11 (.412) | -.02 (.889) |
| OCI-R  Ordering | *h* | .19 (.168) | -.01 (.942) | .19 (.148) | .01 (.950) |
|  | *1-*$\gamma$ | .06 (.647) | -.05 (.753) | .12 (.388) | -.04 (.791) |
|  | $\epsilon$ | -.14 (.284) | -.07 (.622) | -.15 (.269) | -.07 (.586) |

*Note:* OCI-R – Obsessive compulsive inventory -revised. The table suggests that the OCI-R subscales robustly correlating with transition uncertainty were the Neutralizing, Hoarding, Checking and Washing subscales. It should be also noted that the Neutralizing subscales also tended to correlate with the other two parameters, although to a lesser extent. Critically, these exploratory analyses should be taken with caution due to the problem of multiple comparisons, and their exploratory nature. However, they do suggest that transition uncertainty is not exclusively related to checking symptoms. Another robust finding concerns the lack of correlation (i.e. |r's|<.14) with the Obsessing subscale.
