# Supplementary material for: Doubting what you already know: Uncertainty regarding state transitions is associated with obsessive compulsive symptoms
Source: PLoS Comput Biol. 2020 Feb 27;16(2):e1007634. doi: 10.1371/journal.pcbi.1007634 (PMC7046195; doi:10.1371/journal.pcbi.1007634)
Supplement: S2 Table — (DOCX) [file pcbi.1007634.s004.docx]

**S2 Table - Full model comparison results**

**A) Probabilistic condition**

| # | Model | *h* | $\lambda_{0}$ | Response model | WAIC | LOO | WAIC differences in standard error units | | | | | | | | | |
| --- | --- | --- | --- | --- | --- | --- | --- | --- | --- | --- | --- | --- | --- | --- | --- | --- |
|  |  |  |  |  |  |  | 1 | 2 | 3 | **4** | 5 | 6 | 7 | 8 | 9 | 10 |
| 1 | BCP | changing | NA | inv. temp. | 3768.2 | 3770.6 |  |  |  |  |  |  |  |  |  |  |
| 2 | BCP | constant | NA | inv. temp. | 3772.9 | 3774.8 | 0.6 |  |  |  |  |  |  |  |  |  |
| 3 | BCP | changing | NA | ignore $\gamma$ | 3774.8 | 3776.6 | 1.2 | 0.2 |  |  |  |  |  |  |  |  |
| **4** | **BCP** | **constant** | **NA** | **ignore** $\boldsymbol{\gamma}$ | **3775.0** | **3777** | **0.7** | **0.5** | **0** |  |  |  |  |  |  |  |
| 5 | SA | constant | free | ignore $\gamma$ | 3910.2 | 3910.9 | 2.1 | 2 | 2 | **2** |  |  |  |  |  |  |
| 6 | SA | constant | free | include $\gamma$ | 3913.7 | 3914.2 | 2.2 | 2.1 | 2.1 | **2.1** | 0.6 |  |  |  |  |  |
| 7 | BCP | constant | NA | include $\gamma$ | 3924.7 | 3925.1 | 6.8 | 7 | 6.9 | **7.2** | 0.2 | 0.2 |  |  |  |  |
| 8 | BCP | changing | NA | include $\gamma$ | 3943.8 | 3944.5 | 7.2 | 7 | 7.3 | **7.2** | 0.5 | 0.4 | 2 |  |  |  |
| 9 | WSLS | NA | NA | NA | 3991.2 | 3991.8 | 3.3 | 3.2 | 3.2 | **3.2** | 4.3 | 4.1 | 0.9 | 0.6 |  |  |
| 10 | SA | constant | $\lambda_{0}$=0.5 | ignore $\gamma$ | 4014.0 | 4014.5 | 3.7 | 3.6 | 3.6 | **3.6** | 5.7 | 5.1 | 1.2 | 1 | 0.9 |  |
| 11 | SA | constant | $\lambda_{0}$=0.5 | include $\gamma$ | 4051.9 | 4052.2 | 4.5 | 4.4 | 4.4 | **4.3** | 6.3 | 6.4 | 1.8 | 1.5 | 2.3 | 3.2 |

**B) Deterministic condition**

| # | Model | *h* | γ | Response model | WAIC | LOO | WAIC differences in standard error units | | | | | | | | | |
| --- | --- | --- | --- | --- | --- | --- | --- | --- | --- | --- | --- | --- | --- | --- | --- | --- |
|  |  |  |  |  |  |  | **4** | 2 | 1 | 3 | 4d | 2d | 1d | 3d | 5d | 9 |
| **4** | **BCP** | **constant** | **free** | **ignore** $\boldsymbol{\gamma}$ | **1542.6** | **1545.7** |  |  |  |  |  |  |  |  |  |  |
| 2 | BCP | constant | free | inv. temp. | 1548.4 | 1551.6 | **1.5** |  |  |  |  |  |  |  |  |  |
| 1 | BCP | changing | free | inv. temp. | 1577.3 | 1579.7 | **4.2** | 4.7 |  |  |  |  |  |  |  |  |
| 3 | BCP | changing | free | ignore $\gamma$ | 1578.7 | 1581.3 | **5.1** | 4 | 0.2 |  |  |  |  |  |  |  |
| 4d | BCP | constant | $\gamma=1$ | ignore $\gamma$ | 1598.8 | 1600.1 | **2.6** | 2.2 | 0.9 | 0.8 |  |  |  |  |  |  |
| 2d | BCP | constant | $\gamma=1$ | inv. temp. | 1604.9 | 1606.3 | **2.8** | 2.5 | 1.1 | 1.1 | 3.4 |  |  |  |  |  |
| 1d | BCP | changing | $\gamma=1$ | inv. temp. | 1606.1 | 1607.5 | **2.9** | 2.5 | 1.2 | 1.1 | 4.6 | 1.6 |  |  |  |  |
| 3d | BCP | changing | $\gamma=1$ | ignore $\gamma$ | 1640.1 | 1641.1 | **3.9** | 3.6 | 2.3 | 2.4 | 4.5 | 4.1 | 3.9 |  |  |  |
| 5d | SA | constant | fixed | ignore $\gamma$ | 1924.0 | 1924.5 | **7.4** | 7.4 | 6.8 | 6.7 | 5.4 | 5.3 | 5.2 | 4.6 |  |  |
| 9 | WSLS | NA | NA | NA | 1924.4 | 1924.9 | **7.5** | 7.4 | 6.9 | 6.9 | 5.4 | 5.3 | 5.3 | 4.6 | 0.1 |  |
| 5 | SA | constant | free | ignore $\gamma$ | 1925.9 | 1926.4 | **7.5** | 7.4 | 6.8 | 6.8 | 5.4 | 5.3 | 5.3 | 4.6 | 1.1 | 0.3 |

Note: BCP – Bayesian change point model; SA – Bayesian selective attention model; WSLS – Win stay loose shift model; WAIC – widely applicable information criterion; LOO – Pareto smooth importance sampling leave-one-out. Models used to test the main hypotheses (i.e. correlations with OCI-R scores) are highlighted.
