# Supplementary material for: Doubting what you already know: Uncertainty regarding state transitions is associated with obsessive compulsive symptoms
Source: PLoS Comput Biol. 2020 Feb 27;16(2):e1007634. doi: 10.1371/journal.pcbi.1007634 (PMC7046195; doi:10.1371/journal.pcbi.1007634)
Supplement: Attachment — Submitted filename: Rebuttal.docx [file pcbi.1007634.s005.docx]

*Dear Dr. Gershman.*

*We thank you for the time and effort you and the reviewers devoted to the evaluation of our manuscript “Doubting what you knew and checking: uncertainty regarding state transitions is associated with obsessive compulsive symptoms”. We appreciate the interest you and the reviewers have shown in our manuscript, and we value the constructive suggestions and comments, which have helped us clarify and strengthen the manuscript. We first explain the major changes we have made, addressing major and recurring issues raised by the reviewers. Then we respond to all suggestions and comments in a point-by-point fashion.*

*First, we agree with Reviewer 2 that the previous draft might have been too technical. Thus, we have made a substantial revision of the introduction such that it will more clearly introduce the value of the computational model and the intuitive interpretation of its parameters. We have also moved all formulas to the methods section, focusing on intuitive explanations in the results section.*

*Second, we agree with Reviewer 1 that alternative (or additional) explanations for our results should be considered. We thus broadened the discussion to address the relations between our model and other related factors including memory deficits, memory distrust, and intolerance of uncertainty.*

*Third, following the comments of both reviewers we decided to move the analysis and discussion of OCI-R subscales to the Supporting information section. This removes exploratory analyses that are subject to a multiple comparisons problem from the main text, and avoids the questionable suggestion that transition uncertainty is specific to checking symptoms.*

*Finally, we have implemented the additional computational models suggested by Reviewer 1. We also report in greater detail important technical details regarding the Bayesian models used for parameter fitting, and the mixed-effects models. While working on this part of the revision (particularly, reexamining the Stan code), we have noticed that one of the priors used in the previous version of the manuscript was slightly problematic (for a full explanation see our response to point 7 of Reviewer 1). This required us to re-run all models, which required a substantial amount of time, resulting in the relative delay in submitting this revision (we apologize for this delay). However, this did not change the final results substantially. The only meaningful change was that since the correlation between transition uncertainty and OCI-R scores in the deterministic condition became 0.24 instead of 0.26, it was no longer significant, although the difference in effect size is negligible. Overall, we believe that this has improved the validity of our results, and the usefulness of the computational models (which we have now uploaded to OSF) for other researchers. We discuss this choice of priors on pages 39.*

*We now turn to responding to all suggestions in a point-by-point fashion.*

Reviewer #1:

Thank you for the opportunity to review this manuscript, which reports an interesting behavioural study of the association between obsessive-compulsive symptoms and state transition uncertainty in the context of a multi-cue reversal learning task. The study is innovative, methodologically sound, and well-written in general (though see several points below). Strengths of the manuscript include clear and comprehensive presentation of a complex set of computational models, the use of cutting-edge methods for Bayesian model estimation using Hamiltonian Monte Carlo.

*We thank the reviewer for their interest in our manuscript and kind words.*

1. As a general point, given that the data for this manuscript are to be made freely available in an Open Science Framework repository, it would be useful to also provide the Stan code for each model.

*We agree with the reviewer, and have now uploaded the Stan models to OSF -* [*https://osf.io/d6b3m/?view_only=7ec3c823f0b84411af73bca6c4691fd7*](https://osf.io/d6b3m/?view_only=7ec3c823f0b84411af73bca6c4691fd7)

2. As I understand it, the response model for the Bayesian Change Point models assumes that the subject produces a response by marginalising over the different cues (i.e., weighting each cue according to the probability that it is valid). However, this assumption has not been empirically tested. Given that only a single cue is valid on any given trial, it is plausible that participants might respond only according to the cue with the highest probability of being valid (rather than marginalising across all cues). For instance, if my belief is that cue A has a 70% probability of being the valid cue, B a 10% probability, and C a 20% probability, my response might be determined according to the direction of cue A alone (at least in an epsilon-greedy fashion), rather than 0.7 x A + 0.1 x B + 0.2 x C. I would be interested to see this model tested, since it represents a kind of intermediate step between the BCP models (which track the probability of all cues simultaneously) and the SA models (which follow an epsilon-greedy policy for the attended cue).

*We thank the reviewer for this suggestion. We implemented the model the reviewer is referring to (which requires an arg_max statement over p(c|D)) but the sampling from its posterior did not converge (it had many divergent transitions) most likely because such arg_max statements tend to interfere with the gradient evaluation. We thus took a parametric approach, where the amount of maximizing was determined by an additional 'inverse-temperature' parameter, as we explain on pages 30-31:*

*"We sought to examine a maximizing response model, where despite learning about all three cues, the agent responds only in accordance with the most likely cue (rather than averaging across cues). However, introducing an argmax statement impeded the convergence of the model, most likely because such terms often obstruct the smoothness of the posterior. Thus, we took a different approach by introducing an additional 'inverse temperature' parameter β, which controlled the overweighing of the most likely cue in a continuous manner. Specifically, Equation 6 was replaced with:*

| $p\left( R_{t+1} \right)=\left( 1-\epsilon\right)\sum_{c} p\left( R_{t+1} \vert c \right)\frac{{p\left( c \vert D_{1:t} \right)}^{\beta}}{\sum_{c} {p\left( c \vert D_{1:t} \right)}^{\beta}}+0.5\epsilon,$ | ( 8 ) |
| --- | --- |

*Thus, higher β values result in a more maximizing response style, where values close to 1 indicate no overweighting of the most likely cue. Importantly, β had a lower bound at 1, because we did not want this additional parameter to control random or 'no-learning' responding (which was already accounted for by the other parameters)"*

*The results have shown that this model had a roughly similar fit to that of simpler BCP models, and that the posterior estimates for β were relatively close to 1 (median β = 1.67, 95% HDI [1.09, 2.56]), indicating that maximizing is unlikely. In addition, a relatively high correlation (both between participants and between MCMC samples) was found between this β parameter and the γ parameter, complicating the interpretation of parameters. These results are reported in the supporting information section (S2 text):*

*"The best-fitting models in the probabilistic condition (but not the deterministic condition) were models that included an inverse temperature (β) parameter, that were used to examine whether participants use a maximizing response style – i.e., overweighting the most likely cues. However, we decided not to use these models to examine individual differences (i.e. correlation with OCI-R scores) for several reasons. First, the difference between these models and more simple BCP models was negligible (0.7SEs in the probabilistic condition). Second, whereas participants tended to overweight the most likely cue, the magnitude of overweighting was small (median β = 1.67, 95% HDI [1.09, 2.56], where 1 indicates no overweighting, and values > 5 are required for a full maximizing response style). Third, the β parameter had identifiability issues since there was a strong negative correlation (across MCMC samples) between the (group-level) β and the γ parameters (r = -0.87). This tradeoff corresponds with the idea that low γ values can balance maximizing by increasing the variance of* $p\left( c | D_{1:t} \right)$*. That is, for β of 1 (indicating no maximizing) group-level γ was distributed around 0.75 (similar to models not including the β parameter, and to the actual cue validity). Conversely, for posterior β values corresponding with maximizing (~5), group-level γ was distributed around 0.55, which is less appropriate given the actual cue validity in the task. Furthermore, an extremely high correlation was found between the two parameters across participants as well (r = 0.85). This precludes the interpretation of the correlations between each parameter and other variables (e.g., OCI-R scores).*

3. The manuscript claims (e.g., page 18, line 364) that the observed results are specific to OC symptoms, and not symptoms of depression or anxiety, but the evidence for this specificity is weak. As far as I can tell, the specificity claim rests on the finding that the transition uncertainty parameter was significantly correlated with OC symptoms (r = 0.33 and 0.26), but that correlations between the transition uncertainty parameter and depression and anxiety symptoms were not statistically significant (r < .24, p < .1). This evidence is not sufficient to demonstrate that findings are specific to OC, given that, the difference between the statistically significant OC results and the non-significant anxiety and depression results may not itself be statistically significant. To test this claim, it is necessary either to test whether the OC symptoms correlated with transition uncertainty *more strongly* than depression and anxiety symptoms (e.g., using a Fisher r-to-z transformation of the observed correlations), or to show that the OC result was significant even when depression and anxiety were controlled for. If the absence of these findings, there is no strong evidence for the specificity of observed results to OC symptoms, and statements to this effect (e.g., the final sentence of the first Discussion paragraph) should be re-written or removed, and the possibility of confounding by other psychiatric symptoms should be discussed.

*This is a good point. We agree and therefore we have removed these misleading statements from the results and discussion. Furthermore, we now explicitly note these limitations in the results and discussion sections on pages 21:*

*"Transition uncertainty was not significantly correlated with anxious arousal (probabilistic: r = .12, p = .378, deterministic: r = .06, p = .643), depressive symptoms (probabilistic: r = .22, p = .10; deterministic: r = .20, p = .140) or stress (probabilistic: r = -.02, p = .894, deterministic: r = .16, p = .238). Nonetheless, the effect size for depressive symptoms was close to that of OCI-R scores. Examining partial correlations showed that the effect of OCI-R controlling for depressive symptoms was no longer significant (r = .24, p = .075), although it was stronger than the effect of depressive symptoms controlling for OCI-R scores (r = .07, p = .608). Thus, whereas OC symptoms seem to play a larger role here, evidence for specificity is limited, and examination with larger studies is required"*

*And pages 25:*

*"The current study was underpowered to robustly examine the specificity of the effects for OC symptoms (vs. general anxiety or depression). Future studies would benefit from using a large sample allowing to better characterize the contribution of transition uncertainty to different types of symptoms by extracting independent dimensions of psychopathology (e.g., using factor analysis with multiple scales; see* [1]*).*

4. The manuscript states that increased transition uncertainty may explain checking behaviours in OCD. This is a reasonable speculation, but it is important to note that this study only tested this association indirectly. Although the transition uncertainty parameter did correlate positively with self-reported checking, it also correlated equally strongly with symptoms of Neutralizing, Hoarding, and Washing. I understand that the latter analyses were exploratory, but nevertheless it is overstating findings to say that transition uncertainty "can explain excessive checking" (page 21, line 426) given that no specificity to checking behaviours was observed. I feel that this ought to be noted in a revised manuscript. I would also suggest for the same reasons that the "and checking" portion of the manuscript title is a significant overstatement of the results of the manuscript.

*We thank the reviewer for this important comment. We agree that evidence for a specific relationship with checking symptoms is weak. For this reason, and to ameliorate the problem of multiple comparisons inherent to subscale analysis (see response to Reviewer 2), we moved the section focusing on OCI-R subscales to the Supporting information section while stressing its limitation (see Table S1):*

*"Critically, these exploratory analyses should be taken with caution due to the problem of multiple comparisons, and their exploratory nature. However, they do suggest that transition uncertainty is not exclusively related to checking symptoms. Another robust finding concerns the lack of correlation (i.e. |r's|<.14) with the Obsessing subscale.*

*Finally, as suggested by the reviewer we changed the manuscript title to: "Doubting what you already know: uncertainty regarding state transitions is associated with obsessive compulsive symptoms"*

5. As it currently stands, the manuscript tests two families of models: a Bayesian optimal observer family and a family based on the Yu & Dayan approximation. Both are interesting and reasonble, but testing only these two families of models implicitly assumes that participants are not employing some simpler strategy. In order for the results of this manuscript to be interpretable, it is imperative to rule out the possibility that participants are employing a simpler strategy. I would suggest testing a simpler model to rule it out as a competing account of behavioural data: a win-stay lose-switch model that assumes that participants initially pick a cue at random, and then (probabilistically, according to a parameter that varies across participants) stick with that cue if it correctly predicts the outcome, or switch to another cue (again, probabilistically according to a fitted parameter) if their chosen cue does not correctly predict the outcome.

*We thank the reviewer for this helpful suggestion. We have now tested a win-stay lose-switch model against the data (page 15):*

*"****Win-stay lose-shift (WSLS) model.*** *In this simple benchmark model, the agent is assumed to focus on a single cue on each trial. After feedback is obtained, the agent sticks with this cue in case of an expected outcome (i.e. when the target's position matches the orientation of this cue) with probability* $p_{stay}$*, and switches to a different cue in the case of an unexpected outcome with probability* $p_{shift}$*. It is a simple selective attention model that does not require complex Bayesian learning. Since this model shares that same problem of estimating participant's attended cue as the SA model above, a similar solution was used"*

*As depicted in the revised Figure presenting model comparison results (Fig. 4 in the revised manuscript), and in Table S2, the fit of this model was worse than that of the BCP models and most Bayesian SA models.*

6. The manuscript interprets the primary correlation between the transition uncertainty parameter and OC symptoms in terms of increased uncertainty concerning state transitions. According to the model framework presented, the source of this increased uncertainty is a generative model of the environment as more volatile. However, transition uncertainty may have other psychological sources, and it would be useful for the manuscript to discuss these in more depth. For instance, if participants do not maintain Bayesian belief updates but recompute probabilities on the fly, poorer memory recall of previous outcomes would similarly result in an increased reliance on the most recent outcomes. Similarly, lack of confidence in one's own memory for previous outcomes (see, e.g., Boschen & Vuksanovic, Behaviour Research and Therapy, 2007; Tolin et al., Behaviour Research and Therapy, 2001) would lead one to have greater uncertainty about state transitions, but not because one believes that the environment is more volatile. Similarly, in the domain of checking behaviours the manuscript would benefit from engaging with and discussing previous cognitive models of checking that have been proposed in the literature. For instance, it has been proposed (see, e.g., Lind & Boschen; Journal of Anxiety Disorders, 2009; Bennett et al., PLoS Computational Biology, 2016) that checking in OCD results not from increased uncertainty but from increased aversion to uncertainty when it is present, and therefore greater relief from anxiety by checking behaviours.

*We thank the reviewer for this very important comment. We now address the relationship between our results and these alternative (or additional) mechanisms on pages 22-23:*

*"It is important to note that these results do not imply that OCD patients (or individuals with high OC symptoms) necessarily have an explicit belief that the environment is unstable. Indeed, in a recent study only patients' behavior, but not their meta-cognitive beliefs, reflected increased reliance on most recent outcomes* [2]*. Furthermore, increased reliance on recent outcomes might also result, for instance, from poor memory recall, or alternatively lack of confidence in memory. Whereas evidence for poor recall in OCD is scarce* [3]*, distrust in memory has been relatively robust* [3–6]*. However, transition uncertainty might be a mechanism that leads to distrust in memory in OCD: if past states are irrelevant, then predictions based on memory should be regarded as unreliable. Further research is needed to determine to what degree transition uncertainty and memory distrust overlap.*

*Another important consideration concerns the distinction between heightened (transition) uncertainty and an excessive need to resolve uncertainty (i.e. intolerance of uncertainty* [7]*), as both can give rise to excessive information gathering* [7–9]*. Intolerance of uncertainty seems to be supported by a recent study that linked OC symptoms and anxiety to increased information seeking even in a task in which this information had no effect on actual control or performance* [9]*. Future studies should attempt to dissociate the relative contribution of these interacting processes to patients' performance and symptoms."*

7. It would be useful to provide more information on the details of the Stan sampling procedure. How many samples were taken in total, and how many were discarded during the warm-up phase? How many chains were used to sample from the posterior, and did these chains converge (as indiciated by the R_hat statistic)? Were there any divergent transitions in any model?

*We thank the reviewer for this important point. We have added the respective details on page 40:*

*"For each model, the MCMC was run in three chains, with 1000 samples in total, 400 of which were used during warmup to calibrate the Hamiltonian parameters, and were discarded (each of the models required several days to run, and therefore increasing the MCMC samples to considerably larger numbers was impractical). To ensure unbiased sampling, for models in which divergent transitions were detected (typically 1 or 2 transitions), the MCMC acceptance rate parameter (adapt_delta) was gradually increased (up to a maximum of 0.99) – which eliminated all divergent transitions. All models converged as indicated by* $\hat{R}$ *values the did not exceed 1.1. To improve the accuracy of individual-level parameters for the examination of the main hypotheses, the best-fitting models were run again for a larger number of iterations (1500 samples per chain, with 500 warmup samples)."*

*As we notice above, while re-examining the Stan code, we have noticed that one of the priors used in the previous version of the manuscript was problematic. Specifically, the hyper-prior defining group-level variance for all (unbounded) parameters was a wide half-Cauchy distribution with a scale of 2.5. Due to the inverse probit transformations used to convert the unbounded parameters (used for sampling) to the bounded (from 0 to 1) parameters used in the computational model, this wide variance inadvertently resulted in bimodal priors for the bounded parameters (with modes near the bounds). Since this does not correspond with our intention to specify a uniform prior for participant-level parameters, we changed the prior for the group-level variance parameters in all models to a half-t distribution with a scale of 0.2, and a v parameter of 50. This prior balanced the goal of resulting in uniform priors for participant-level parameters, with that of not constraining participant-level parameters to be too similar to each other. Specifically, the use of 0.2 as a scale eliminated the bi-modal participant-level prior distribution (indeed, a similar value was used in the hBayesDDM package* [10]*). Conversely, the use of the half-t distribution (with v=50; instead of a half-normal distribution, which was used in the hBayesDDM package, for example) allows for some variation between participants even in the case in which group-level mean parameter are close to 1 (as was the case in some parameters).*

**Minor comments**

Abstract: The acronym 'OCD' is not introduced here or elsewhere.

*We now define this acronym upon first usage in both the abstract and manuscript.*

Page 5, lines 96-97: Given that the Yu & Dayan model is rather complex (with links to attention, learning, and neuromodulation, as the manuscript notes), it might clarify things to expand on precisely which of its features have not been empirically examined.

*We now state more explicitly that we examine only the part of the model pertaining to Bayesian learning (and not neuromodulation, for example) on page 7:*

*"Since this is the first empirical investigation of this task, we also examined whether the approximate Bayesian learning model suggested by Yu and Dayan* [11] *accounts better for participants' performance than an optimal Bayesian model"*

Page 5, lines 105-106: Citations should be given for the attribution of this "common preconception" regarding OCD.

*We have now added a citation to this idea (see pages 4-5, lines 86-88)*

Page 6, line 117: I believe that "which allows to independently quantify" is ungrammatical. "which allows us to independently quantify" would be correct, as would "which allows independent quantification of".

*We thank the reviewer for this correction.*

Page 6, line 129: The exact information concerning the timing of the shift is not provided until the Methods section, but it would be useful for the reader in interpreting the results presented in this section to know that there was only one shift per block, in roughly the middle of the block.

*We now state explicitly in the results section (page 8):*

*"Each condition included 88 trials, with a single shift occurring after either 40 or 48 trials (counterbalanced across participants and conditions)."*

Page 7, Figure 1: '40/48' somewhat implies that the transition occurred after either 40 or 48 trials. 40-48 would be more accurate.

*As we now explicitly say on page 27 (lines 580-683, and in our response above, the shift occurred after either 40 or 48 trials:*

*"for half the participants the probabilistic condition included 40 trials before the shift and the deterministic condition included 48 trials before the shift, whereas for the other half of participants this was flipped"*

Page 7, section beginning line 142: I feel that it would be useful for the reader to know more basic behavioural descriptive statistics before the manuscript jumps directly to the inferential analyses. For instance, on what proportion of trials did participants respond correctly? How did this change over the course of the pre-shift and post-shift blocks? Were participants at asymptote at the time the shift occurred, and if so, what was the asymptotic level of performance?

*We added a basic descriptive section on page 9 and a new figure depicting learning curves (Figure 2)*:

*"Overall, participants' mean accuracy in the deterministic condition was 0.94 and 0.93 pre and post-shift. Mean accuracy in the probabilistic condition was 0.79 and 0.78 pre- and post-shift. As depicted in Figure 2, participants reached an asymptote (of ~0.97) quickly in the deterministic condition, with performance dropping immediately after the shift. Conversely, in the probabilistic condition, participants' mean performance was more unstable pre-shift, with decreases likely reflecting incorrectly interpreting probabilistic errors as real contingency shifts. A more stable increase in performance was observed post-shift, likely reflecting a belief that a shift has already occurred, such that accumulated knowledge appeared more reliable."*

Page 7, line 147: The acronym 'OCI-R' is used before it is defined.

*We have corrected the sentence to say:*

*"[…]to examine the effects of OC symptoms as measured by the Obsessive Compulsive Inventory-Revised (OCI-R* [12]*) on performance."*

Page 12, line 256: Typo - Criterion, not Criteria.

*We thank the reviewer for this correction*

Page 15, line 15: Several questions about these confidence intervals: What percentage confidence interval is presented in this figure? Are the confidence intervals across subjects or trials? Is this a Bayesian highest density interval (in which case, it would be more appropriate to call it a credible interval) or a frequentist confidence interval? If the latter, what is the rationale for mixing Bayesian and frequentist statistics? I applaud the manuscript's efforts to present an estimate of uncertainty around model fit statistics, but I think a little more transparency would be useful.

*We thank the reviewer for this helpful comment. Originally we used the standard errors as reported by the loo package* [13] *to compute confidence intervals (i.e. +/- 2*SE). The reason behind this was to have a scale-free measure of how large the difference between the fit of two models is. However, following the reviewer's comment, we re-examined the validity of this analysis. Whereas more empirical work is needed, it has been suggested that the variance estimates for model fit indices currently used in the loo package may underestimate the true standard error (see* [*https://discourse.mc-stan.org/t/interpreting-elpd-diff-loo-package/1628*](https://discourse.mc-stan.org/t/interpreting-elpd-diff-loo-package/1628)*;* [14]*). Furthermore, SEs of individual models cannot be used to infer the SEs of the* ***differences*** *between two models (due to dependence). Thus, we removed SEs for individual models from the manuscript, and have generally decided to refrain from writing that two models have a significantly different fit.*

*Regardless, in Table S2 (Supporting information) we present the differences between models in WAIC SE units (such that a difference of 1 corresponds with a WAIC difference that of 1 SE; with SEs extracted from the loo package). This provides a scale-free measure for the comparison of two models, while avoiding making assumptions regarding the sampling distribution of these indices. As we say there, these estimates can be used to infer that two models have a* ***similar*** *fit, but not that two models are significantly different (because of the possibility for underestimation). See S2 text in the supporting information:*

*"The rightmost part of the tables presents the WAIC differences in standard error units. These can be used to estimate whether two models are statistically different from each other. For example, a difference that is 2SEs or larger indicates that a classical confidence interval (±1.96SE) will not include zero. However, since studies suggest that SE estimates as those used by the loo package* [13] *can underestimate the true variance* (up to a factor of 2; [14])*, we refrain from categorizing model differences as significant. However, a difference that is less than 2SEs can be used to infer that the corresponding models are not significantly different than each other* (since overestimation is unlikely)*"*

*In addition, we calculated weights (Akaike weights for the WAIC, and pseudo-BMA weights for the PSIS-LOO) for the fit indices presented in Figure 4 in the manuscript to provide an additional means for model comparison, as we now state on page 16:*

*"To support the interpretation of these results, these values were used to approximate the relative likelihood of each model being the best model by calculating model weights (Akaike weights for the WAIC, and pseudo-BMA weights for the PSIS-LOO* [15]*)."*

*And in the caption of Figure 4:*

*"The size of the triangles represent the relative weights (i.e. approximation of the relative likelihood) of the different models."*

Page 20, line 403: Typo - "this task differ".

*We thank the reviewer for this correction*

Page 22, line 463: "After a random number of 40-48 trials". I assume that integers were chosen from this range according to a uniform distribution? It would be helpful to have that information explicitly reported in the manuscript.

*We corrected this sentence in accordance with our response above (page 27):*

*"After a random number of either 40 or 48 trials (counterbalanced, such that for half the participants the probabilistic condition included 40 trials before shift and the deterministic condition included 48 trials before the shift, whereas for the other half of subjects this was flipped), a contingency shift (hereafter “shift”) occurred"*

Page 22, line 463: At the switch-point, was the new predictive cue guaranteed to be different from the previously predictive cue, or was the new predictive cue equally likely to be any of the three cues? I assume the former, but it would be helpful to have this information explicitly reported in the manuscript (especially since the models appear to assume the latter).

*This is an important point. We now highlight the word "different" to stress the fact that after a shift a different cue became relevant. We discuss the reasons for this inconsistency between the task and the models on page 29:*

*"* *Note that whereas in the model a switch is followed by a uniform distribution over all three cues, in the task the same cue is never resampled after a switch. The reason for defining h in this way is that we focused on the uncertainty regarding state transitions rather than the probability for a change in contingencies (i.e. volatility). Thus, for example, h =1 corresponds with completely discounting previous knowledge (whereas in a completely volatile environment previous knowledge can be used to infer which cue is irrelevant)"*

Page 34, lines 700-701. "All models included a random intercept and a random slope when relevant". Did all models converge? If a model did not converge, what protocol was used for simplifying the model? Also: given that the lme4 package does not produce p-values, please provide information on which approximation was used to estimate these.

*As we now state explicitly on page 41, all models converged. The majority of models were logistic GLMM models, for which the lmer package does provide p-values (because such models use a Z statistic, which does not require the computation of degrees of freedom). To calculate significance for models with a continuous response variable (analyses pertaining to the prediction of information-theoretic measures of prediction errors) we used the lmerTest package which uses the Satterthwaite degrees of freedom, as we now state explicitly on page 41.*

Supplementary material, Table S2: According to what criterion were models inferred to be significantly different from one another (alphabetical superscripts)?

*Originally, we used the SE values for each pairwise comparison to calculate confidence intervals (+/- 2*SE). However, as we say in our response above, after studying the subject further, we decided to refrain from suggesting significant differences, and instead report the differences in SE units, while explicitly stating what can and what cannot be inferred from them in the supporting information (S2 text).*

Reviewer #2: This is a timely and important paper. It employs a Computational Psychiatry approach to elucidate mechanisms behind clinically observed symptoms of obsessive-compulsive disorder (OCD). Emergent literature converges in understanding that there are significant impairments in decision making among individuals with OCD even in contexts unrelated to their main symptomology. Including computational modeling approach in broader investigations is a more effective strategy to explore such impairments than clinical observations alone, since it allows testing empirically hypotheses that are largely based on clinical intuition. The results are interesting and are with many implications for further research.

*We thank the reviewer for their interest and encouraging feedback.*

The major challenge of papers like this is to make the tools and results more accessible to clinical audience. The current draft falls a bit short of that. It is a VERY technical paper. It is not an easy read for people without computational background. I suggest this is a main goal for the next revision.
Maybe, state two alternative hypotheses (H0: Reduced transition uncertainty, Ha: Inflexibility in strategies) very clearly from the start and explain how clinical observations may support each of them. Then list all relevant quantitative variables with clear intuitive explanation of what they mean, and how using these variables may test H0 against Ha. This will also reinforce the comparative strength of using computational approaches – an ability to empirically test alternative hypotheses. But limitations (and how they may relate to the assumptions) should be discussed as well.

*We thank the reviewer for this important feedback. Following the reviewer's suggestion we have made a substantial revision of the introduction such that it will more clearly introduce the added value of the computational model and the intuitive interpretation of its parameters. Following the reviewer's suggestion we also more explicitly contrast our hypothesis with the inflexibility hypothesis, while outlining the specific computational predictions of both. The revised introduction can be found on pages 4-7 in the manuscript (and is not cited here due to its' substantial length).*

*We also discuss the limitations of our computational approach in the discussion (pages 25-26):*

*"The results of this study depend on the validity of the chosen BCP model, and on the assumption that people use some sort of Bayesian inference in this task. Whereas we examined several different Bayesian and non-Bayesian computational models, other alternative models could still be imagined. The behavioral pattern of increased reliance on more recent feedback can obtain a different theoretical meaning in computational models that make different assumptions (e.g., a reinforcement learning model with a 'forgetting' parameter* [16]*)."*

I think that the models should not be described in such details in the main text and could be left *more* for Methods Section. But what is needed is an added value of this approach, what is predicted and can be tested in very simple intuitive terms, why it is important to use multiple models to test these predictions, and why it is important to have both probabilistic and deterministic conditions – again, a value added in a very plain language.

*Following the reviewer's suggestion, and to make the manuscript more readable for clinical audiences we have removed all equations from the results section, and instead attempted to better explain the differences between the different models, and the meaning of their parameters verbally (see the above response, and pages 12-15). Furthermore, in addition to the major revision of the introduction (see response above), we now explicitly state the importance of comparing different computational models on page 13 (lines 249-250):*

*"This stage is crucial for determining whether participants actually follow a Bayesian model when solving the task.".*

Then present all results in the simple language as well – consistent with this hypothesis and rejects this hypothesis + exploratory analyses (maybe some of the exploratory results could be moved to supplements – there are so many of them, may be try to prioritize?). This or another strategy to present the results in more clinically relevant manner is likely to improve the impact of this research.

*We thank the reviewer for highlighting the need to better contextualize the results. We have added a brief summary of the computational results on page 19 (lines 381-384):*

*"These results show that underperformance related with OC symptoms is indeed the result of under-weighing accumulated knowledge, and not of perseveration or non-specific stochasticity in the response process"*

*We also explain further (page 19) the importance of examining the two feedback processing variables (i.e. surprisal and model updating; having moved the respective equations to the methods section):*

*"As outlined above, transition uncertainty and observation uncertainty interact in determining the weight given to feedback (i.e. prediction errors). Thus, this computational setup allowed us to use participants' best fitted parameter values to estimate two trial-level measures of the processing of feedback in the probabilistic condition. First, we examined how unexpected each outcome was to participants (using a measure of surprisal; see Equation 9). For example, the case in which all three cues point to one direction but the target appears at the other direction is highly unexpected. Second, we examined the degree to which each outcome made the participants change their beliefs about the relevant cue (using the KL-divergence; see Equation 10). Crucially not all unexpected feedback leads to learning. Indeed, in the example given above, the unexpected target provides no new information regarding the relevant cue. More generally, high transition uncertainty increases both measures, whereas observation uncertainty increases surprisal but decreases model updating. At the extreme case wherein* $\gamma=.5$*, feedback is always unpredictable, yet is completely uninformative about the relevant cue. Thus, examining the feedback processing measures can help better understand the* ***interaction*** *between the two uncertainty parameters in high OC participants. Furthermore, these two, partially dissociated types of prediction error* [17] *have different neural markers* [17–21]*"*

*Finally, we accept the reviewer's suggestion of distinguishing exploratory analyses from analyses directly aimed at examining our hypothesis (and the competing perseveration hypothesis). Thus all analysis regarding OCI-R subtypes were moved to the Supporting information section (see response below)*

There is a LARGE number of correlational results reports in the paper. This raises a concern of the multiple comparisons problem. Significance levels are unlikely to survive the corrections. Furthermore, the data appears to be subject to increasing variance (vs. symptom severity) and not necessarily normally distributed. Maybe it is more appropriate to use nonparametric correlational analyses here. Yet, the more powerful approach would be to modify the model to allow individual variations in key parameters (e.g. hi ~ h * OC-R). This would resolve both concerns. Would it be possible to fit such model given the data?

*We thank the reviewer for this important suggestion. First, we would like to emphasize that all correlational analyses were non-parametric as stated on page 41:*

*"Correlations between fitted parameters (taking the median of each posterior distribution) and participants' scores on the OCI-R and the DASS-21 were tested by using a permutation test, due to the violation of the normality assumption for most variables* [22]*".*

*Previously this appeared only in the methods section, and thus, we now added a brief mention of this in the results section (page 18, lines 374-375):*

*"a non-parametric permutation test due to the violation of normality"*

*Furthermore, since we had no prior knowledge regarding the precise form of these relationships we preferred to use a non-parametric approach rather than entering OCI-R scores into the computational models (which by-definition requires a parametric approach).*

*We agree that multiple comparisons might be a problem, particularly in the context of analyzing OCI-R subscales. Therefore, and because our main hypothesis was related to OC symptoms in general, we moved the analysis pertaining to subscales to the Supporting information section for interested readers, where we also highlight that:*

*"these exploratory analyses should be taken with caution due to the problem of multiple comparisons, and their exploratory nature" (Supporting Information, page 4)*

**Minor:**
p. 10. “where (+1|,1:) reflects the prior probability that the previously relevant cue is no longer relevant on trial t+1 (i.e., transition uncertainty).”
- you probably meant “the probability that this experience is still/no longer relevant (i.e., in case of a changepoint)”, as you correctly state on p. 24.

*We thank the reviewer for this correction. Since we removed all formulas from the results section, only the correct statement (in the methods section) has remained.*

p. Fig. 3 legend “additional evidence for negligent, chance-level performance.”
- performance based exclusion criteria should be stated before the data/results. The way it is presented it sound post hoc.

*One of the benefits of having an explicit parameter measuring random responding is the ability to detect participants who did not pay attention/did not understand the task, establishing a strong criterion for exclusion. Critically, this decision is not related to the actual hypotheses tested in the paper. We now state this explicitly on page 13:*

*"The probability for random responding was parameterized by* $\epsilon$ *(very low values of this parameter can also be used to indicate negligent performance, which justifies exclusion)"*

p. 21. “The next step is to develop a more ecological design, examining whether transition uncertainty can explain actual OC symptoms.”
- this sounds contradictory to the presented motivations (general cognitive impairments) and correlational analyses with OC severity. Probably meant to test the model in clinically relevant contexts/symptom provocation paradigms.

*We thank the reviewer for this comment. We have rephrased this sentence to say:*

*"The next step is to develop a more ecological design, examining the role transition uncertainty plays in clinically relevant contexts"*

p. 21. “Next, we examined the specificity of transition uncertainty to OC symptoms. In support of specificity, transition uncertainty was not significantly correlated with anxious arousal, depressive symptoms or stress…”
- Need to include the rationale for this before data are presented. As is it shows up from nowhere.

*We thank the reviewer for this comment. We now introduce the 'Specificity to OC symptoms' section by saying (page 21):*

*"Finally, we sought to examine whether transition uncertainty is related specifically to OC symptoms, as hypothesized, or whether this relationship can be accounted for by general distress, anxiety or depression."*

1.	Gillan CM, Kosinski M, Whelan R, Phelps EA, Daw ND. Characterizing a psychiatric symptom dimension related to deficits in goaldirected control. eLife. 2016;5: 1–24. doi:10.7554/eLife.11305

2.	Vaghi MM, Luyckx F, Sule A, Fineberg NA, Robbins TW, De Martino B. Compulsivity Reveals a Novel Dissociation between Action and Confidence. Neuron. 2017;96: 348–354.e4. doi:10.1016/j.neuron.2017.09.006

3.	Olley A, Malhi G, Sachdev P. Memory and executive functioning in obsessive-compulsive disorder: a selective review. Journal of affective disorders. 2007;104: 15–23. doi:10.1016/j.jad.2007.02.023

4.	Hermans D, Engelen U, Grouwels L, Joos E, Lemmens J, Pieters G. Cognitive confidence in obsessive-compulsive disorder: Distrusting perception, attention and memory. Behaviour Research and Therapy. 2008;46: 98–113. doi:10.1016/j.brat.2007.11.001

5.	Tolin DF, Abramowitz JS, Brigidi BD, Amir N, Street GP, Foa EB. Memory and memory confidence in obsessive–compulsive disorder. Behaviour Research and Therapy. 2001;39: 913–927. doi:10.1016/S0005-7967(00)00064-4

6.	Boschen MJ, Vuksanovic D. Deteriorating memory confidence, responsibility perceptions and repeated checking: Comparisons in OCD and control samples. Behaviour Research and Therapy. 2007;45: 2098–2109. doi:10.1016/j.brat.2007.03.009

7.	Lind C, Boschen MJ. Intolerance of uncertainty mediates the relationship between responsibility beliefs and compulsive checking. Journal of Anxiety Disorders. 2009;23: 1047–1052. doi:10.1016/j.janxdis.2009.07.005

8.	Bennett D, Bode S, Brydevall M, Warren H, Murawski C. Intrinsic valuation of information in decision making under uncertainty. PLoS computational biology. 2016;12: e1005020.

9.	Bennett D, Sutcliffe K, Tan NP-J, Smillie LD, Bode S. Anxious and obsessive-compulsive traits are independently associated with valuation of non-instrumental information. BioRxiv. 2019; 768168.

10.	Ahn W-Y, Haines N, Zhang L. Revealing neurocomputational mechanisms of reinforcement learning and decision-making with the hBayesDM package. Computational Psychiatry. 2017;1: 24–57.

11.	Yu AJ, Dayan P. Uncertainty, neuromodulation, and attention. Neuron. 2005;46: 681–692. doi:10.1016/j.neuron.2005.04.026

12.	Foa EB, Huppert JD, Leiberg S, Langner R, Kichic R, Hajcak G, et al. The Obsessive-Complusive Inventory: Development and validation of a short version. Psychological Assessment. 2002;14: 485–495. doi:10.1037//1040-3590.14.4.485

13.	Vehtari A, Gelman A, Gabry J. Practical Bayesian model evaluation using leave-one-out cross-validation and WAIC. Stat Comput. 2017;27: 1413–1432. doi:10.1007/s11222-016-9696-4

14.	Bengio Y, Grandvalet Y. No Unbiased Estimator of the Variance of K-Fold Cross-Validation. Journal of Machine Learning Research. 2004;5: 1089–1105.

15.	Piironen J, Vehtari A. Comparison of Bayesian predictive methods for model selection. Statistics and Computing. 2017;27: 711–735.

16.	Niv Y, Daniel R, Geana A, Gershman SJ, Leong YC, Radulescu A, et al. Reinforcement Learning in Multidimensional Environments Relies on Attention Mechanisms. Journal of Neuroscience. 2015;35: 8145–8157. doi:10.1523/JNEUROSCI.2978-14.2015

17.	Barto A, Mirolli M, Baldassarre G. Novelty or Surprise? Frontiers in Psychology. 2013;4: 1–15. doi:10.3389/fpsyg.2013.00907

18.	Seer C, Lange F, Boos M, Dengler R, Kopp B. Brain and Cognition Prior probabilities modulate cortical surprise responses : A study of event-related potentials q. Brain and Cognition. 2016;106: 78–89. doi:10.1016/j.bandc.2016.04.011

19.	Schwartenbeck P, FitzGerald THB, Dolan R. Neural signals encoding shifts in beliefs. NeuroImage. 2016;125: 578–586. doi:10.1016/j.neuroimage.2015.10.067

20.	Kobayashi K, Hsu M. Neural Mechanisms of Updating under Reducible and Irreducible Uncertainty. The Journal of Neuroscience. 2017;37: 6972–6982. doi:10.1523/JNEUROSCI.0535-17.2017

21.	Nassar MR, Bruckner R, Frank MJ. Statistical context dictates the relationship between feedback-related EEG signals and learning. BioRxiv. 2019; 581744.

22.	Bishara AJ, Hittner JB. Testing the significance of a correlation with nonnormal data: Comparison of Pearson, Spearman, transformation, and resampling approaches. Psychological Methods. 2012;17: 399–417. doi:10.1037/a0028087
